# Intravital imaging of hair-cell development and regeneration in the zebrafish

**DOI:** 10.3389/fnana.2013.00033

**Published:** 2013-10-11

**Authors:** Filipe Pinto-Teixeira, Mariana Muzzopappa, Jim Swoger, Alessandro Mineo, James Sharpe, Hernán López-Schier

**Affiliations:** ^1^Centre for Genomic Regulation (CRG)Barcelona, Spain; ^2^Universitat Pompeu Fabra (UPF)Barcelona, Spain; ^3^Institució Catalana de Recerca i Estudis Avançats (ICREA)Barcelona, Spain; ^4^Unit Sensory Biology and Organogenesis, Helmholtz Zentrum MünchenMunich, Germany

**Keywords:** hair cells, auditory, zebrafish, intravital fluorescence microscopy, regeneration, lateral line system, development

## Abstract

Direct videomicroscopic visualization of organ formation and regeneration *in toto* is a powerful strategy to study cellular processes that often cannot be replicated *in vitro*. Intravital imaging aims at quantifying changes in tissue architecture or subcellular organization over time during organ development, regeneration or degeneration. A general feature of this approach is its reliance on the optical isolation of defined cell types in the whole animals by transgenic expression of fluorescent markers. Here we describe a simple and robust method to analyze sensory hair-cell development and regeneration in the zebrafish lateral line by high-resolution intravital imaging using laser-scanning confocal microscopy (LSCM) and selective plane illumination microscopy (SPIM). The main advantage of studying hair-cell regeneration in the lateral line is that it occurs throughout the life of the animal, which allows its study in the most natural context. We detail protocols to achieve continuous videomicroscopy for up to 68 hours, enabling direct observation of cellular behavior, which can provide a sensitive assay for the quantitative classification of cellular phenotypes and cell-lineage reconstruction. Modifications to this protocol should facilitate pharmacogenetic assays to identify or validate otoprotective or reparative drugs for future clinical strategies aimed at preserving aural function in humans.

## Introduction

**Figure 1:**
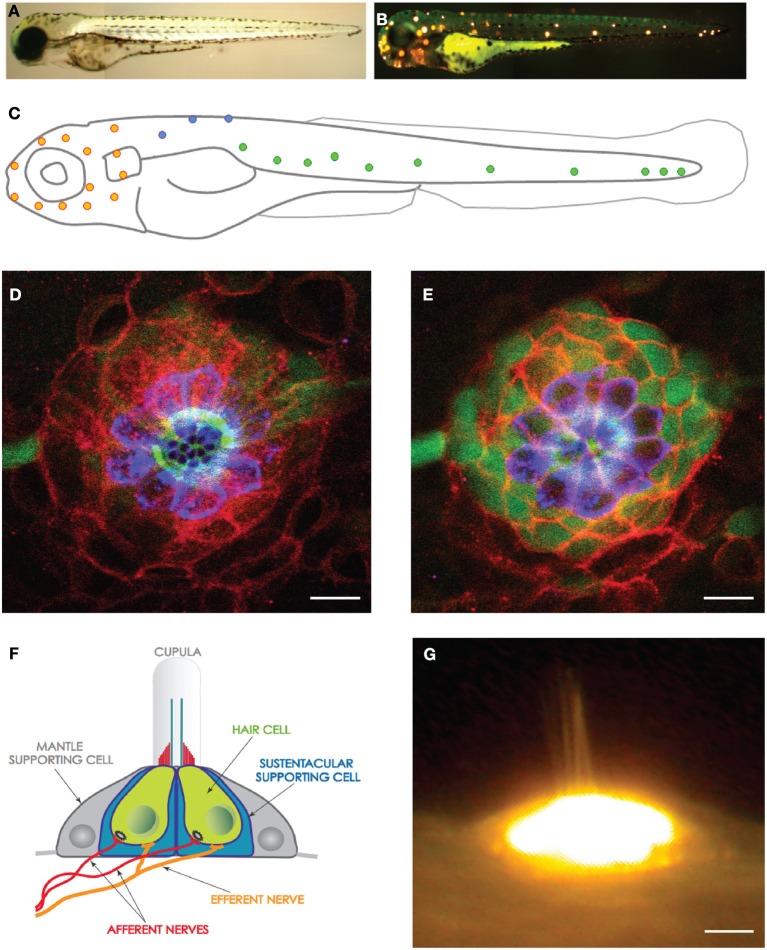
**The lateral line and neuromasts in the zebrafish larva. (A)** Low magnification view of a zebrafish larva under transmitted light. **(B)** Hair cells in the lateral line were labeled with the fluorescent vital dye DiAsp (bright orange). It shows the superficial distribution of neuromasts along the anterior (head) and posterior (trunk and tail) lateral-line systems. **(C)** Schematic representation of the distribution of the neuromasts in the lateral line of a zebrafish larva. Orange highlights the anterior neuromasts, green the posterior neuromasts, and blue the dorsal neuromasts. **(D,E)** High magnification confocal image of a frontal view of a posterior neuromast revealing the hair cells (blue), supporting cells (red and green). **(F)** Schematic representation of a neuromast viewed from the side, depicting every known cell, including the neurons. **(G)** High magnification side-view image of a mature neuromast labeled with the vital dye DiAsp. Scale bars are 10μm.

**Figure 2:**
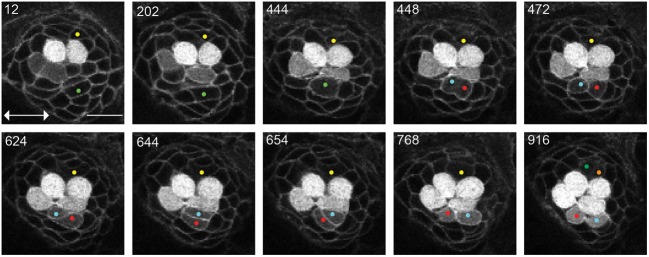
**UHCPs division and planar cell inversion**. Frames from Supplementary Movie 1 at the time-points indicated A 916′ min series of confocal images of a double-transgenic Tg[Cldnb:mGFP;SqET4] regenerating neuromast. Two prospective UHCPs were identified retrospectively by playing the time series backwards and labeled with a yellow and green dot. Each UHCP eventually divides into a pair of sister hair cells, labeled in Cyan/Red and Green/Orange, respectively. On minute 624′ the sibling hair cells in the lower part of the neuromast begin to rotate around their contact point, eventually breaking the line of mirror symmetry. This complete inversion of the sister hair cells restores the line of mirror symmetry realigning the cells along the neuromast axis of planar polarity (double-headed arrow in the first panel). All images correspond to a single focal plane with the cells of interest in focus. Scale bar is 10 μm.

Direct *in toto* visualization of organ formation and repair is a powerful strategy to study cellular processes that often cannot be replicated *in vitro* (Megason and Fraser, 2007; Pittet and Weissleder, 2011; Rompolas et al., 2012). A general feature of high-resolution intravital imaging is its reliance on the optical isolation of cells or sub-cellular structures in the whole animal by the transgenic expression of fluorescent markers (Faucherre et al., 2010; Dempsey et al., 2012). The formation of specialized organs during embryonic development involves the activation of genetic programs that determine cell fate, cell proliferation, and the assembly of tissues into functional organs. Animals spend the overwhelming majority of their lives as adults, when many organs are under acute or chronic physical or toxicological stress and can sustain damage. Most organisms have evolved two general strategies to maintain organ function throughout life. The most prevalent is protection by isolating organs into body cavities. Although effective, isolation is not optimal for sensory systems because their external components need to be exposed to the environment to fulfill their function. Therefore, organisms have evolved a second strategy to maintain sensory abilities over long periods: regeneration and repair. Paradoxically, however, mammals have lost the capacity to repair the sensory elements of the inner ear (Corwin and Oberholtzer, 1997; Kelley, 2003; Kros, 2007; Edge and Chen, 2008; Brigande and Heller, 2009; Rubel et al., 2013). If damaged by infection, drugs or stress, the ear's mechanosensory hair cells cannot be replaced, leading to irreversible loss of hearing and balance (Collado et al., 2008). The resulting sensory dysfunction develops into a major handicap that dramatically decreases the quality of life of the affected individuals. Deafness often causes acute communication difficulties, which represents a significant socioeconomic problem considering that it afflicts more than 10% of the population in industrialized countries (Magilvy, 1985). According to the British charity “Deafness Research UK,” 1 in 7 people in the United Kingdom suffer from hearing impairment. Notwithstanding the biological and clinical interest of deafness and balance disorders, the mechanisms that underlie hair-cell homeostasis remain poorly understood because of the inherent difficulty to study the inner ear in animals after birth. By contrast, aquatic vertebrates such as the zebrafish have a superficial and anatomically simple “hearing” system called lateral line (Figures 1A–C) (Raible and Kruse, 2000; Ghysen and Dambly-Chaudiere, 2007; Behra et al., 2009). This mechanosensory system detects hydromechanical variations around the animal body and serves to command behaviors such as schooling, rheotaxia, prey capture, and obstacle and predator avoidance (Dijkgraaf, 1963; Ghysen and Dambly-Chaudiere, 2007; Bleckmann and Zelick, 2009). Fishes of lotic and lentic freshwater ecosystems have a well-developed superficial lateral-line system, whose functional units are called neuromasts (Figures 1D–G). The superficial lateral line is very accessible to experimentation and imaging. The neuromast is a volcano-shaped epithelium that projects from the animal's body surface, into the water (Figure 1F). A mature neuromast is 20 μm in height and has a diameter of 50 μm. It is formed by around 60 cells, of which one third are hair cells and the remainder divided between peripheral mantle cells and central sustentacular (supporting) cells (Figures 1D,E). Importantly, identically to their mammalian counterparts, hair cells in the zebrafish are susceptible to ototoxicity or mechanical damage (Harris et al., 2003; Chiu et al., 2008; Behra et al., 2009; Brignull et al., 2009). However, unlike mammals, fishes can regenerate hair cells rapidly and orderly after damage (Williams and Holder, 2000; Harris et al., 2003; López-Schier and Hudspeth, 2006; Behra et al., 2009). Our group was the first to unambiguously identify resident hair-cell progenitors, and to study how their dynamic relationship with supporting cells maintains a constant, functional organ throughout the animal's life (López-Schier and Hudspeth, 2006). Our results have revealed that hair cells in neuromast are always born sequentially and in pairs along a single axis (Figure 2) (López-Schier and Hudspeth, 2006). These observations allowed us to predict the compartmentalization of the neuromast epithelium. We subsequently confirmed this prediction using the SqET4 transgenic zebrafish line that identified resident hair-cell progenitors, and the SqET20 line that revealed a cellular territory where hair-cell progenitors generate at high frequencies (Wibowo et al., 2011). Thus, the detailed intravital *in vivo* analyses have unveiled two previously unknown cell types in neuromasts. Here we detail a simple, fast and inexpensive protocol to characterize hair-cell development and regeneration by *in toto* high-resolution live imaging. This protocol has been optimized during over 10 years of experience using the zebrafish and high-resolution imaging (López-Schier and St. Johnston, 2001; López-Schier et al., 2004; López-Schier and Hudspeth, 2006; López-Schier, 2010; Swoger et al., 2011; Wibowo et al., 2011). It relies on the stable expression of engineered transgenes coding for fluorescent proteins in specific cellular populations of the lateral line. The procedure only requires a small zebrafish colony and access to a Laser-Scanning Confocal Microscope (LSCM) for image acquisition (Wibowo et al., 2011). We also describe the handling and mounting of samples to enable imaging by Selective Plane Illumination Microscopy (SPIM) (Huisken et al., 2004; Greger et al., 2007; Huisken and Stainier, 2009; Swoger et al., 2011; Kaufmann et al., 2012). Finally, this approach requires a desktop computer equipped with free or inexpensive commercial software for qualitative or quantitative image analysis and rendering.

### Rationale

Hair-cell development is likely regulated by mechanisms that are cell-type specific, and controlled by a complex activation of signaling pathways in both hair-cell progenitors and supporting cells (López-Schier, 2004). Notwithstanding its major scientific and clinical significance, the mechanisms that underlie the normal production of hair cells remain poorly understood. Studies aimed at characterizing the behavior of endogenous hair-cell progenitors should be carried out in animal model systems with accessible neuroepithelia and capable of regenerating hair cells efficiently. Pharmacological treatments that produce deafness in mammals are also lethal to hair cells in the zebrafish. Hair cells in the zebrafish lateral line begin to regenerate less than 12 h after loss, to eventually produce a complete anatomical and functional recovery of the organ in less than 72 h. Many important questions remain unanswered: What is the source of newly formed hair cells? How is the process of hair-cell regeneration controlled? How the three-dimensional architecture of the epithelium recovers? Results from experiments in several species suggest that hair cells regenerate from supporting cells by trans-differentiation. Other results indicate that supporting cells re-enter mitosis before producing new hair cells. Although supporting cell trans-differentiation may be a simple mechanism to replace damaged hair cells, supporting-cell amplification is also required because animals need to maintain the number and proportion of the different cell types over many years. Thus, cell proliferation is eventually essential for neuroepithelial homeostasis. An attractive possibility is that the neuroepithelium harbors a population of pluripotent progenitor cells dedicated to regenerate hair cells (Harris et al., 2003; Huisken et al., 2004; Rivolta et al., 2006; Oshima et al., 2009, 2010). This hypothesis has taken some impulse from a series of results suggesting the existence of stem cells in the utricle. These cells could be characterized further for potential clinical applications. Stem-cell-based therapies represent one promising avenue to repair damaged inner ears (Brigande and Heller, 2009; Chen et al., 2012; Rivolta, 2013). However, they require invasive surgical procedures and post-operative follow-up. The shortcomings of cell replacement therapy suggest that mobilizing resident progenitor cells to replace damaged tissues is a viable alternative with therapeutic potential (Mizutari et al., 2013). This protocol provides a simple and robust method to obtain information about the molecular profile, localization, and behavior of progenitor cells within a mature organ.

### The zebrafish as an experimental model system

The zebrafish (*Danio rerio*) is a small tropical fish whose biology has been extensively characterized. Interesting developmental and behavioral aspects of the zebrafish have made it a favorite research organism in genetics, biomedicine, and drug discovery for the past three decades. It compares favorably with other experimental animal model systems, offering the following advantages:

- It has a rapid life cycle. Adult pairs produce thousands of progeny economically and several generations per year. This permits quantitative/statistical analyses, and facilitates the recovery of offspring carrying multiple mutations and transgenes.
- The embryo develops rapidly, externally, and is optically transparent, which enables easy visualization of organs for detailed cellular and physiological analyses (Kemp et al., 2009).
- The large-scale generation of transgenic zebrafish is technically simple, and the maintenance of large colonies for experimentation and high-throughput drug screening is feasible even in single-laboratory settings (Murphey and Zon, 2006; Detrich et al., 2011).
- Alternatively, engineered genes can be expressed transiently in the zebrafish by DNA or RNA injections or electroporation, facilitating, and speeding functional studies by gain- or loss-of-gene function. A trained person can inject up to 500 eggs, or generate up to three transgenic lines per day (Xu et al., 2008).
- Unlike *Drosophila, C.elegans* or the mouse, many organs in the zebrafish regenerate after damage.
- Because all vertebrates share many of the cellular and physiological mechanisms that underlie how organs develop and perform, the zebrafish provides a highly relevant model to human biology and disease, as has been demonstrated by numerous studies (Santoriello and Zon, 2012).
- The lateral line organ combines the three-dimensional organization of a sensory receptor with the possibility of systematic experimental intervention, offering a simple model whose dynamics can be visualized *in vivo* over long periods and under controlled physiological conditions.
- The extensive collection of reagents for experimentation has significantly improved the capabilities of this system (Moens et al., 2008; Faucherre and López-Schier, 2011; Kettleborough et al., 2011), and reinforced the general belief that the lateral line is an ideal model to study the basic mechanisms that control sensory-organ development and regeneration.

## Methods

### Handling of the zebrafish

The success of these procedures crucially depends on the proper handling and mounting of the zebrafish embryo or larva. Sample handling needs to be practiced to ensure animal survival. We routinely monitor the animal's vital signs after imaging (heart beating and blood flow). If samples will be further processed for immunohistochemistry staining after live imaging, they should be fixed as soon as possible after imaging ceases.

### Obtaining zebrafish larvae for experiments

Zebrafish are maintained in E3 Embryo medium. To make an E3 medium stock solution (60×) dissolve 172 g NaCl, 7.6 g KCl, 29 g CaCl_2_.2H_2_O and 49 g MgSO_4_7H_2_O in 10 L of distilled H_2_O (dH_2_O). To avoid water spoilage add methylene blue (Sigma-Aldrich; cat. No.M4159). Make a methylene blue stock solution of 4 mgml^−1^ in water. For a final working solution, add 70 μl of stock solution to 1 L of E3 Embryo medium. Fish used were maintained under standardized conditions and experiments were performed in accordance with protocols approved by the PRBB Ethical Committee of Animal Experimentation.

### Necessary equipment for raising fish and collecting eggs

Divisible acrylic tanks for zebrafish breeding and plastic strainers for egg or larva collection. Incubator set at 28.5°C to maintain embryos and larvae at constant temperature. An epifluorescence-equipped stereomicroscope for initial screening of experimental animals. In addition, disposable 6 or 12 well culture plastic plates, Pasteur plastic pipettes will be needed.

### Hair-cell ablation in zebrafish larvae

A stock solution of Neomycin 500 mM (neomycin trisulphate salt hydrate, SIGMA-Aldrich N6386) should be maintained at 4°C, and diluted in E3 medium without methylene blue to a working concentration of 250 μM. The stock solution should be kept in a 1.5 ml Eppendorf tube sealed with Parafilm and saved in an Eppendorf box to minimize the risk of contamination. It is important to be cautious with the use of neomycin. Mechanosensitive hair cells, including those of humans, are extremely sensitive to aminoglycosides. Neomycin is quickly absorbed by the body surface, therefore, gloves and protective goggles should be used during all the steps that involve the use of neomycin.

### Components for mounting zebrafish larvae

To anesthesize zebrafish, prepare a stock solution of tricaine (MS222 or MESAB: 3-amino benzoic acidethylester) at 25×. Dissolve 0.4 g Tricaine, in 97.9 mL of dH_2_O. Adjust the pH to ~7 by adding 9.1 mL of 1 M Tris (pH 9). Store at 4°C. Make one percent low–melting point agarose in E3 to mount embryos for observation. In an Erlenmeyer flask make 50 ml of 1% LMP agarose 436 (Bio Rad, Cat. 161-3112) by adding 500 mg of agarose to 50 ml 437 of E3 medium without methylene blue. Heat the solution for 2 min in a microwave at maximum power to dissolve the agarose. If small aggregates are still found agitate the solution and heat up to obtain a clear solution. The solution will be very hot. Use a temperature protective gear to handle the Erlenmeyer flask at this stage. Transfer the solution to a 50 ml Falcon tube and store in a water bath at 42°C. Let the agarose cool down to 42°C before using it. This agarose can be used for one week. For confocal microscopy, hair loops are appropriate to manipulate the zebrafish larvae on cover-glass-bottomed culture dishes. For SPIM imaging, 100/200 μL glass capillaries (cat. no. 701910) and piston rods (cat. no. 701936) from Brand GmbH.

### Observation and analysis of hair-cell development and regeneration

A fluorescence stereomicroscope equipped with an external light source for fluorescence excitation and appropriate filter set for GFP and RFP detection can be used for screening the samples to be imaged.

For confocal imaging, a microscope equipped with lasers for GFP (488 nm), RFP (543 nm) excitation and preferentially with a motorized stage to allow multi-position imaging.

For SPIM imaging, the microscope can be equipped with lasers operating at 488 nm (for excitation of GFP) and 543 nm (RFP), and the appropriate emission filters. Excitation power and wavelength are computer-controlled via an Acousto-Optic Tunable Filter (AOTF); the filter wheel for changing emission filters is also computer controlled. Typically water-dipping objective lenses are used for detection, ranging from 20 × /0.5 to 63 × /0.9, depending on the desired resolution and field of view. Illumination is with air objectives, e.g., a 10 × /0.3. Detection is performed with a CCD or sCMOS camera (in this work we assume a Hamamatsu Orca-ER CCD camera). The sample is mounted on a computer-controlled mechanical stage with 3 spatial and one rotational degrees of freedom (the rotation axis is perpendicular to the illumination and detection axes). See Greger et al. (2007) for a technical description of a SPIM microscope similar to the one used in the present work (Greger et al., 2007). We routinely use the public domain ImageJ/Fiji software for image processing.

### Identification of transgenic expression of fluorescent proteins in the zebrafish lateral line

Collect the zebrafish larvae to be screened in a Petri dish with 50 ml of methylene-blue-free E3 medium with tricaine (250 μM). Screen and select embryos with a stereomicroscope using the appropriate light filter and select those larvae expressing fluorescence in the lateral line neuromasts. If the larvae to be analysed are younger than 3 dpf they have to be dechorionated. We do not recommend dechorionation with pronase as this decreases the survival rate. We favour manual dechorionation. This can easily be achieved with the help of a pair of microsurgical forceps. Hold the chorion with one forceps and with the other forceps grip the chorion and tear it. It is important to minimize the exposure of the larvae to tricaine. If the larvae are not to be treated with neomycin for the next hours transfer them to fresh methylene-blue-free E3 medium without tricaine.

### How to identify transgenic larvae used in this study

**Table:** 

Tg[SqEt4] fish	The SeqEt4 line strongly expresses the green-fluorescent protein (GFP) in the cytoplasm of neuromast hair cell, and weakly in the unipotent hair-cell progenitors (UHCPs) (Parinov et al., 2004; Choo et al., 2006; Wibowo et al., 2011). GFP is also weakly expressed in the skin (periderm), which makes the identification of SqEt4 larvae straightforward by fluorescent light under low-magnification stereomicroscopy.
*Tg[Cldnb:mEGFP; SqET4] fish*	In the *Tg[Cldnb:mEGFP*] a membrane-localized green-fluorescent protein (EGFP) is expressed in all the cells of the migrating primordium, and in all the cells of the neuromasts and interneuromast cells. In this line mEGFP is also strongly expressed in the olfactory placode (Haas and Gilmour, 2006). The identification of *Tg[Cldnb:mEGFP; SqET4]* larva should be done by the strong expression of GFP in the olfactory placode and the weak expression of GFP in the fish skin.
*Tg[Cldnb:mGFP; atho1a:tdTomato] Histone2B.GFP cRNA injected fish*	The *Tg[atho1a:tdTomato*] line expresses a red-fluorescent protein in the cells of the neuromast that activate atho1a expression, including UHCPs and young hair cells (Wibowo et al., 2011). *Histone2B.GFP* marks every nucleus in the embryo and larva.

### Neomycin induced hair cell ablation

This step takes about 3 h. In a 100 ml Erlenmeyer flask prepare 50 ml of E3 medium without methylene blue with neomycin (250 μM) by adding 250 μl of the neomycin stock to 50 ml of E3 medium. Carefully agitate the Erlenmeyer to mix and transfer the neomycin solution from the Erlenmeyer to a 50 ml Petri dish. Additionally prepare one Petri dish with 50 ml of E3 medium without methylene blue. Collect the larvae in a 50 ml Petri dish with E3 medium without methylene blue with tricaine (250 μM). A critical step is to keep the larvae in E3 medium with methylene blue until the desired developmental stage and transfer them to methylene-blue-free E3 medium at least 2 h before the neomycin treatment, as methylene blue affects the survival of the larvae upon treatment. With the help of a plastic Pasteur pipette transfer the larvae with the minimum amount of medium to the Petri dish with the neomycin solution. Carefully disperse the larvae in the plate by gently squeeze the Pasteur pipette. To collect the larvae hold the Petri dish on the bench and move it horizontally in very small circles until the larvae gather centripetally in the center of the petri dish. Another possibility is to use the Pasteur pipette to promote flow of the medium. This can be done by placing the tip of the pipette parallel and next to the inside border of the petri dish and gently squeeze. With the help of a clean plastic Pasteur pipette transfer the larvae with the minimal amount of medium to the previously prepared petri dish with 50 ml of E3 medium without methylene blue and let the larvae recover for at least 2 h. Some larvae will eventually die due to the neomycin treatment. These larvae should be removed from the medium.

Mechanosensitive hair cells, including those from humans, are extremely sensitive to neomycin. Use gloves throughout these steps.

### Mounting of larvae for live confocal imaging

**Figure 3:**
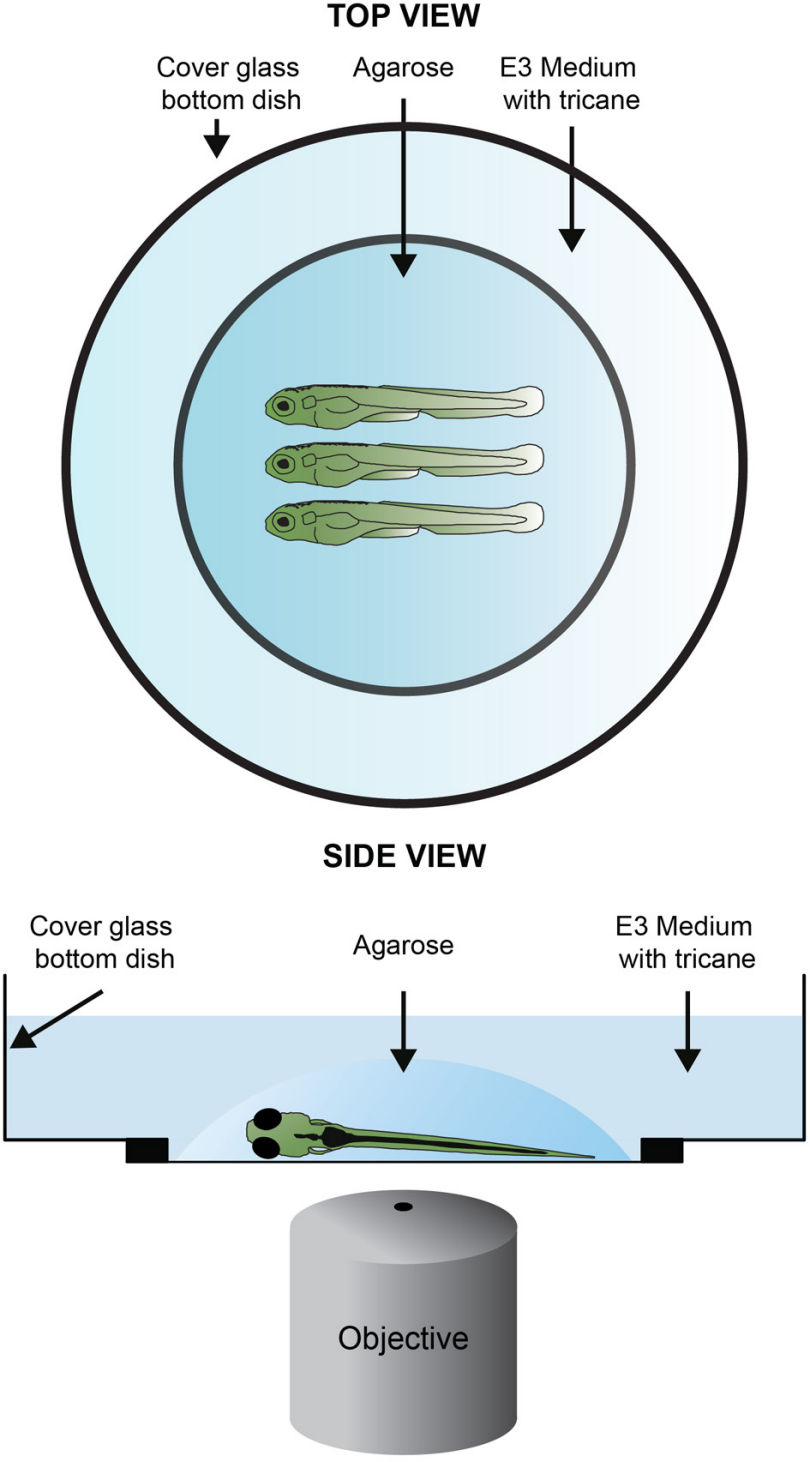
**Sample mounting for confocal imagin**. One to four larvae are, laterally and equally oriented, placed in the center of a cover glass bottom dish. One drop or two of 1% LMP (low–melting point) agarose in E3 are added covering the larvae. If needed readjust the position of the larvae using an embryo loop. Once the agarose solidifies, the embryos are covered with methylene-blue-free E3 medium containing tricaine and the lid is placed on top of the dish.

In a 1.5 ml Eppendorf tube prepare 1 ml of 1% LMP agarose with tricaine by adding 60 μl of the tricaine stock solution to 940 μl of the agarose solution. Keep the Eppendorf at 37°C in a bench thermomixer. Transfer the zebrafish larvae directly to a cover-glass-bottomed culture dish. With a plastic Pasteur pipette release a drop of the agarose solution to cover the larvae. Carefully rotate the larvae with a hair loop so that the desired side to image faces the bottom side of the holder. The larvae should be mounted all in the same orientation and in the center of the dish (Figure 3). This step is essential if the imaging needs to be done at the early stages of neuromast development. No more than 3 larvae should be mounted per culture dish to maximize survival rate. Wait for a few minutes to let the agarose solidify (~35 min at room temperature). When solidified, the agarose becomes a translucent and hard gel, which can be tested by touching it with the hair loop. At this stage carefully fill the dish with 2.5 ml of E3 medium without methylene blue and tricaine (250 μM). Cover the dish with its lid.

### Mounting of larvae for live SPIM imaging

**Figure 4:**
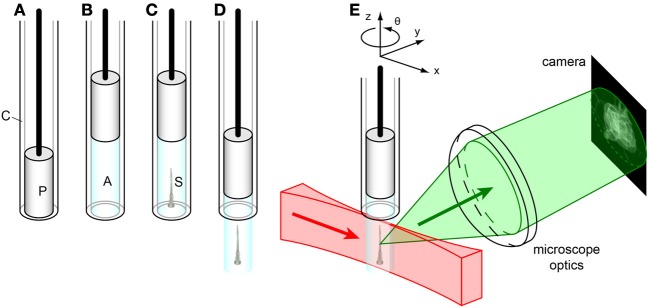
**Sample mounting for SPIM imaging. (A)** Glass capillary (C) with plunger (P). **(B)** The plunger is pulled back, drawing liquid agarose (A, cyan) into the capillary. **(C)** Before the agarose solidifies, the sample (S) is carefully injected into the liquid agarose in the capillary by using a p20-pipette and a yellow tip cut in the end. **(D)** After the agarose solidifies, the plunger is pushed down, extruding the agarose plug containing the sample from the capillary. **(E)** The sample is mounted on a *xyz*θ positioning stage in the SPIM. The laser light sheet (red) illuminates the sample along the *x*-axis, and the detected light (green) is collected orthogonally (along the *y*-axis) by the microscope optics to form an image on the camera.

In a 1.5 ml Eppendorf tube prepare 1 ml of 1% LMP agarose with tricaine by adding 60 μl of the tricaine stock solution to 940 μl of the agarose solution. Keep the Eppendorf at 37°C in a bench thermomixer. Prepare a p20 pipette with a yellow tip by cutting 0.5 cm from the end, so that the hole is big enough for the larvae to enter. Place the capillary on the surface of the liquid agarose and pull the plunger back until the capillary is filled with 1 cm of agarose. (Figure 4). Immediately take the anesthetized larvae in E3 medium with the p20 pipette and carefully inject it in the capillary with the minimal amount of liquid that is possible. Lay the capillary horizontal for a few minutes to let the agarose solidify (10 min at room temperature). Mount the capillary in the SPIM device and fill the sample chamber with methylene-blue-free E3 medium with tricane.

### Imaging setup for confocal microscopy

This step takes about 1 h. To minimize photobleaching, evaporation of the medium and “burning” the larvae, the lasers should be set to the lowest settings that allow a sharp visualization of the neuromasts. As a guideline, in a Leica SP5 system laser configuration window set the Argon laser power to 30%. In the laser power adjustment window located in the beam path screen set the laser power intensity between 13% and 19%. Place the dish covered with its lid in the appropriate support. Place the dish so that the larvae are horizontally aligned in the computer acquisition screen. Locate the neuromast with a low magnification objective (20×). Use the microscope oculars to screen the larvae and identify potential neuromasts of interest. Save their location in the software location panel. From this moment, it is crucial not to touch the dish. A typical experiment allows the imaging of 4 neuromasts with a 2-min acquisition time. We recommend to save the location of 8–10 neuromasts at this pre-screening stage.

### Neuromast identification

**Figure 5:**
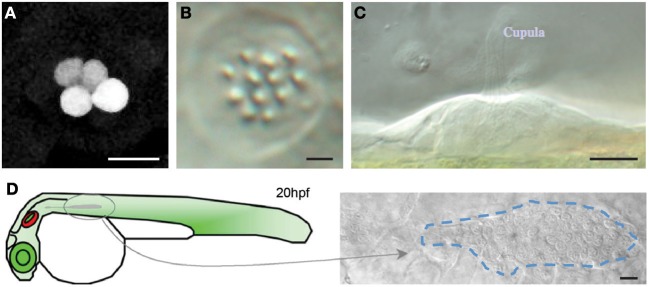
**Neuromast identification (A) *Tg[SeqEt4]* upon neomycin treatment, typically 1–4 hair cells can be found in the neuromasts. (B,C)** Top **(B)** and lateral **(C)** brightfield view of a neuromast. **(D)** Scheme depicting primordium migration in a 20 hpf larvae and top brightfield view of a migrating primordium, identifiable as a group of ~100 cells just under the epidermis. Scale bars are 10 μm.

The identification of a neuromast in the *Tg[SeqEt4]* upon the neomycin treatment, can typically be done by the presence 1–4 hair cells (Figure 5). These correspond to hair cells that were not mature during the treatment, and were therefore insensible to neomycin. During lateral line development, when a neuromast is deposited a UHCP is typically dividing giving rise to two hair cells. In both cases the presence of these hair cells makes the identification of the neuromasts easier, even with a 20× objective. However, if the researcher wishes to image neuromasts where no hair cells are present, the screening has to be done using a 40× objective. In this case favor the use of *Tg[Cldnb:mGFP; SqET4]* larvae where neuromast identification can be easily done by the expression of *Tg[Cldnb:mGFP]*. The identification of neuromasts without hair cells during regeneration in the *Tg[SeqEt4]* is significantly more difficult. In this case the neuromast has to be identified using bright field light. As a guideline, neuromasts are typically located at the somite boundaries, in the middle of the myoseptum where the chevron shifts orientation (Figure 5). If the researcher wants to image the first pair of hair cells being produced it is necessary to start the time series when a neuromast is being deposited. The researcher should then identify the migrating primordium, which is easily identifiable as a group of ~100 cells just under the epidermis (Figure 5).

Change to a higher magnification lens (40×). For long imaging sessions an Oil Immersion objective must be used to avoid evaporation. Adjust the pixel configuration to 512 × 1024. If imaging has to be done at the early stages of neuromast development it is essential that larvae are positioned with the lateral line aligned along the long axis of the field of view, because the neuromasts are still being deposited and can move significantly during the first hours of the time lapse. Proper orientation of the lateral line avoids having the neuromasts moving out of the field of view as they are deposited. Using the previously saved locations screen the samples and identify the neuromasts you want to image. Delete undesired locations. A typical experiment allows the simultaneous imaging of 4 neuromasts.

Adjust the Z-stack for the first location. Use existing hair cells or *Tg[Cldnb:mGFP]* expression to identify the Z-stack limits. Set up the Z-stack so that it contains the entire extent of the neuromast. The Z-stack should start on the tip of the hair cells and end in the bottom part of the neuromast. As default use a step size of 2 μm. If using the *Tg[Cldnb:mGFP]* with the purpose of cell tracking do not use a step size bigger than 2 μm. Because hair cells develop/regenerate the neuromast assumes its typical volcano shape and its volume changes as it grows in size. This, and the continuous development of the larvae, might originate movement of the region of interest out of the Z-stack boundaries during data acquisition. To avoid this add an extra 2 μm to the boundary that sets the beginning of the stack and 5 μm to the boundary that sets the end of the stack on the bottom of the neuromast/hair-cells. If the system does not allow specific Z-stacks for individual positions use as general stack boundaries the tip of the hair cells/neuromast that is closest to the objective and the bottom of the neuromast/hair-cell that is furthest away from the objective. If imaging a *Tg[SeqEt4]* larvae where no hair cells are present use the skin of the animal as a reference to set the beginning boundary and from there set a ~20 μm stack. Adjust the position of the neuromast to the center of the field of view and resave the position with the new settings. Repeat this procedure for each position to be imaged.

Adjust the Gain settings. To visualize UHCP's that weakly express GFP, set up the gain so that the peak GFP signal from existing hair cells is just above the saturation threshold. If imaging neuromasts without hair cells use hair cells of another neuromast in the sample as a reference. Activate the Bidirectional Scanning option to double the scanning speed, run a trial scan and judge the quality of the images. Adjust the acquisition time and length according to the needs. If the acquisition time is less than the total time between the start of consecutive time-points, it might be ideal to apply an increased number of averaging passes to improve image quality. For live imaging it is always recommended to use an average between lines to avoid any image disturbance due to movement of the tissue. Save the LIF file and then save each time-lapse as individual TIFF series with the name “STACKNAME_STACK TIFFS.” Create a logbook with the following details of each time lapse: Transgenic line used, duration of time lapse, Z-stack step size, Z-stack number of steps.

### Imaging setup for SPIM

The primary advantage for live-sample imaging that SPIM has over confocal microscopy is the reduced photo-damage of the sample. In confocal microscopy, for each plane that is imaged in the sample the entire volume is illuminated, and sectioning is achieved by rejecting out-of-focus fluorescent emission with a confocal pinhole. In contrast, in SPIM only the in-focus plane of the sample is illuminated, so no out-of-focus light is generated and all of the emitted fluorescence can be used. This means that SPIM provides more information per unit of exposure of the sample. One can therefore achieve higher signal-to-noise ratios in SPIM than would be possible in confocal imaging without excessive photo-bleaching or damage. Similarly, because of the lower photo-toxicity, with SPIM one can image more often and over longer periods of time.

The following steps assume a custom-built SPIM instrument similar to that described in Greger et al. (2007). Although SPIM induces substantially less photo-damage than confocal imaging, it is still advisable to minimize over-exposure of the sample. One typically begins with the camera gain set to maximum and the laser power ~10% of maximum. If the resulting signal-to-noise ratio is insufficient, the gain can be reduced and/or the laser power increased. Before imaging, align the SPIM so that the light sheet coincides with the focal plane of the detection arm, and adjust the light sheet so that it is as narrow as possible, but of uniform thickness over the camera's field of view. In our custom-built SPIM, this is done by adjusting a gimble mirror and adjustable slit that were built into the illumination path for this purpose. However, the procedure will vary between different implementations of SPIMs. Insert the sample with the region of interest to be imaged facing approximately toward the detection objective lens. See Swoger et al. (2011) for a discussion of how to optimally orient the sample. Using transmission contrast, try to center the approximate region of interest in the field of view of the camera. Depending on the developmental stage of the neuromasts, this may not be possible in transmission, but at a minimum it should be possible to find the myoseptum. This will save time and exposure of the sample when working in fluorescence. Switching to fluorescence contrast, use the sample translation stage to locate and center a neuromast in the field of view. Determine the limits of the axial scanning as described for confocal imaging. Typical slice spacing is 1–2 μm. Save the positioning parameters to define the scan for that neuromast. Translate the embryo to locate another neuromast and repeat these procedures until about 4 scans have been defined. The exact number that can be done will depend on the number of slices in each stack and the maximum time between repeated stack acquisitions. Set parameters for each desired channel: emission filter, laser wavelength and intensity, and camera gain and exposure. Multiple channels can be acquired in plane-interlaced mode. The parameters for each channel should be selected to optimize image quality while imaging a typical neuromast “live.” Set the timing parameters: temporal spacing of the sequential acquisitions, Δ*t*, (typically 2–5 min) and total time of the experiment (anywhere from minutes to days, as desired). Δ*t* will be limited by the time it takes to scan each neuromast and the number of neuromasts scanned. Before beginning the full time-lapse, it can be helpful to do a single scan of all of the neuromasts, in order to determine the minimum value of Δ*t*. If this turns out to be longer than the desired Δ*t* it will be necessary to reduce the required time by, e.g., reducing the number of neuromasts imaged. See also the “The acquisition time is too long” section under Troubleshooting, below. Choose a filename prefix and destination folder, and start the time-lapse acquisition.

During the initial 2 or 3 h of the scanning, occasionally check that the neuromasts are each still centered in their fields of view. Because the agarose is not a completely rigid gel it can relax slightly when the sample is first put into the microscope, resulting in a shift of the region(s) of interest. This is especially important when imaging freshly deposited neuromasts, because these can still move relative to the surrounding tissue even if the agarose is stable. Any drifts can be corrected “on-the-fly” by updating the position parameters in the software user interface.

### Data processing

Open the TIFF series in ImageJ/Fiji. Convert stack to Image5D using the Image5D Plugin. When requested use “Z” for 3rd dimension and “Time” for 4th dimension. Fill in the details of the time lapse according to your log book. Project the Z-stack to a single plane using the project Z-stack option from the Image5D plugin. To add a time counter, use the Time stamper plugin. Fill in the details of the time lapse according to your log book. Save the file as a TIFF file. To make a movie save the file as AVI and choose an appropriate frame rate for your needs. As a reference a 12 h long time lapse with an acquisition time of 2 min takes 36 s to play with a speed of 10 frames per second. The timing of data processing ranges from ~15 min to 6 h per dataset.

### If processing a data set obtained using *Tg[Cldnb:mGFP; SqET4]* with the purpose of cell tracking

Open the TIFF series in ImageJ/Fiji and reverse the stack using the reverse tool in Image/Stacks/Tools. Save the LIF file as Image Sequence in a designated folder with the name “STACKNAME_Reversed STACK TIFFS.” It is crucial to tick the option use slice labels as file names. You have just inverted the stack so that the last time point of the acquired data is now the first to be displayed and the first time point is the last to be displayed. CRUCIAL! Remove the scale of the stack in Analyze/Set Scale. Convert stack to Image5D using the Image5D Plugin. When requested, use “Z” for 3rd dimension and “Time” for 4th dimension. Fill in the details of the time lapse according to your log book. Open the MtrackJ plugin. In the displaying menu tick the option “Display only track points at current time” and reduce the “track width” to 0. Identify the cell(s) to be tracked. Press ADD in the MtrackJ panel and with the mouse cursor over the cell of interest press the mouse button. The program will save the details (XYZT coordinates) of the selection and move to the next time index after adding the point. If the selection is no longer visible on the current Z-axis use the Z-axis scroll bar in the Image5D window to relocate the region of interest. Repeat these steps until the end of the time lapse. Save the tracking using the saving function In the MtrackJ control panel. In the MtrackJ control panel press the Measure Button. Observe that the measurement window shows for each time point the details of the selection (XYZT coordinates). Save the measurement results as an excel file. If more cells will be tracked, restart MtrackJ to clear the memory, go to the first image of the stack and identify the cell to be tracked. Repeat the described procedure for each desired tracking.

**Figure 6:**
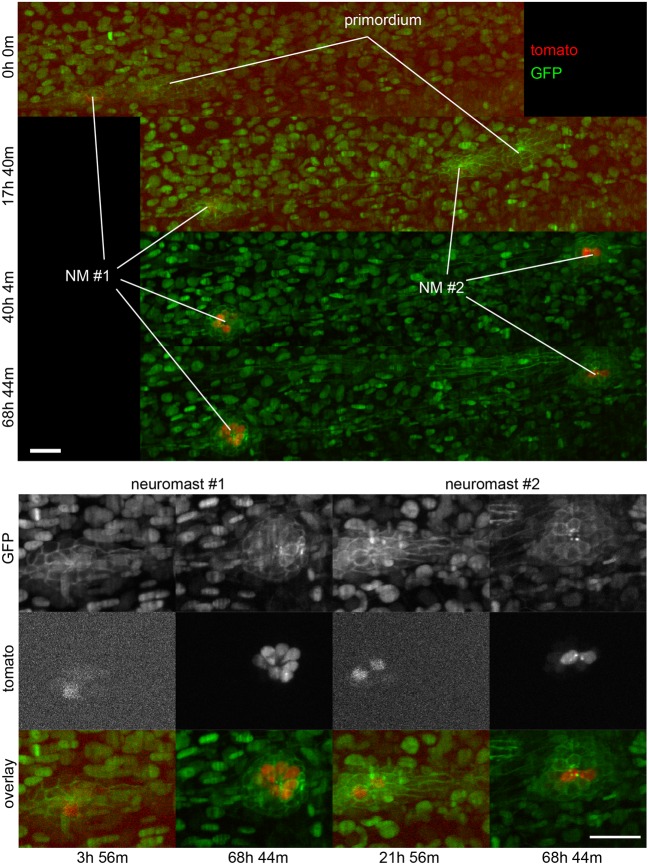
**Three-dimensional imaging by SPIM**. Frames from Supplementary Movie 2 at the time-points indicated. **Top**, full field of view. **Bottom**, selected regions of interest containing neuromasts 1 and 2. A 2-channel 68 h time-lapse recorded using SPIM, showing the deposition of 2 neuromasts and their subsequent development. Ath1 (showed in red) is expressed in hair-cells and can be detected in hair-cell progenitors a few hours before the neuromasts are deposited. ath1:RFP is observed later in dividing, young and mature hair-cells. All the cell nuclei are stained in green (H2B:GFP RNA) and the lateral line system is highlighted in green with the Cldnb:mEGFP reporter. All images are maximum-value projections along the detection axis. The brightness of each image has been normalized independently, so that the tdTomato fluorescence at early time-points is visible. Scale bars are 25 μm.

Open the folder “STACKNAME_Reversed STACK TIFFS” where the stacks were saved as an Image sequence. Observe that each file name contains a reference to the time point and a Z coordinate. Using the details of the selection (XYZT coordinates) in the excel file select for each time point the file corresponding to the Z coordinate of the selection. If tracking more than one cell use only the Z coordinates of one of the cells for the selection. The selected files should be copied to a new folder named “STACKNAME_Z_SELECTION.” You have just created a time lapse of the dataset that follows the cell(s) of interest. Open the files in “STACKNAME_Z_SELECTION” as image sequence in ImageJ. Open MtrackJ. Load the previous saved tracking results. To show the tracks along all time points, in the MtrackJ control panel Displaying options select a Track width size of 3 pixels. If do not want the number of each MtrackJ point to be shown in the MtrackJ control panel under the section Displaying/Font size choose 0 pixels. Crop the time lapse to the region of interest. For this use the rectangular selection tool to define the region of interest and then use the function crop in Image/Crop. Reverse the reversed time lapse using the reverse tool in Image/Stacks/Tools. You have just inverted the time lapse so that the first time point of the acquired data is now the first to be displayed and the last time point is the last to be displayed. To add a time counter, use the Time stamper plugin. Fill in the details of the time lapse according to your log book. This is the final TIFF file. Save it with the appropriate name. To make a movie from the file save it as an AVI and choose an appropriate frame rate for your needs. As a reference a 12 h long time lapse with an acquisition time of 2 min takes 36 s to play with a speed of 10 frames per second (Figures 3, 6).

## Troubleshooting

### The acquisition time is too long

The acquisition time can be reduced by:

1. Reducing the amount of neuromasts imaged.
2. Increasing the Zstack step size.
3. Reducing the amount of line averaging passes.
4. Acquiring more than one neuromast in the same microscopy field.

### The fish die during image acquisition

1. Check the concentration of the anesthetic and eventually reduce it to a minimum of 0.7X
2. Laser power is too high and you are “frying” the fish. Reduce the intensity of the lasers.
3. Too many larvae in the dish. Reduce the number of larvae to be imaged.

### The data set does not open in image J

If using a 32 bits operating system, the maximum amount of memory that can be allocated in ImageJ is 1.7 GB. By default, ImageJ sets the memory limit to 2/3 of physical memory (640 MB maximum) the first time it is run. Use the Edit > Options > Memory command to make more than the default amount of memory available to ImageJ. Note that setting the “Maximum Memory” value to more than about 75% of real RAM may result in poor performance due to virtual memory “thrashing.” Make sure the to tick the option “Use virtual stack,**”** which only reads one image plane into memory at a time, which is essential to open datasets that are too large. If after following the ImageJ Wiki instructions you still cannot open the data set:

1. Before saving the original tiff files use the cropping tool in the Leica Software to reduce the size of the image to the region of interest.
2. If the data set is still too big to open in ImageJ divide the TIFF series in batches of a maximum size of 1.7 GB. Save each batch in an individual folder with the name “STACKNAME_STACK TIFFS_BATCH_XX,” where XX is the number of the batch. Do not separate the data of one time point between batches.
3. Perform the data processing of each batch as described previously. Save each new file as “STACKNAME_BATCH_XX,” where XX is the number of the batch in a folder named “STACKNAME_BATCHES.”
4. Open the files in “STACKNAME_BATCHES” as an image sequence in ImageJ. This is the final TIFF file. Save it with the appropriate name.

## Anticipated results and concluding remarks

### Advantages and limitations of intravital imaging in the zebrafish lateral line

Key advantages:

- The main advantage of using live imaging in whole living zebrafish to study hair-cell regeneration in the lateral line is the possibility of following every cell over very long periods at very high resolution in the normal cellular environment.
- Alterations of gene function by targeted mutagenesis or transgene expression in the zebrafish enables the easy study of gene function.
- Hair-cell ablation with aminoglycoside drugs is simple, effective, and does not cause unnecessary stress to the animals during treatments because there is no need for injections or anesthesia. Hundreds of animals can be treated simultaneously.

Main limitations:

- Confocal and SPIM imaging remains relatively expensive.
- The study of a large number of zebrafish by long-term live imaging for statistical analyses is a long and tedious procedure.
- These protocols are not likely to be affective in fish older than 4 weeks. However, if older fish are necessary, imaging can be performed on neuromasts located in the animal's caudal fin.

### Intravital imaging in the zebrafish larva

The powerful techniques available in the zebrafish render it an excellent model system to study normal physiology or disease onset and progression at the level of genes, cells, and whole organ systems. Genetically encoded fluorescent sensors are a powerful tool to visualize cellular structures dynamically *in vivo/in toto* in wild type and genetically- or pharmacologically-modified specimens. One of the most important reasons for developing an *in vivo/in toto* analysis is that it will provide a remarkably accurate and rapid assay for screening factors that control of hair-cell development and regeneration in the natural context.

### Other applications of the protocol and future uses

Mechanosensory hair cells serve to detect sound and to sense head movements and acceleration. Hearing and balance are complex processes that allow organisms to react appropriately to relevant environment stimuli. Because hair cells are exclusive to vertebrates, their development, function, and regeneration cannot be studied in other classical experimental model systems such as the fruit fly or the roundworm. Currently, hundreds of cell type-specific Gal4 driver lines are available for the zebrafish, making it possible to unambiguously define cell types, cell status, and to perform genetic mis-expression studies. In addition, marking cells with genetically encoded fluorescent proteins will enable the sorting of such cells after dissociation, for transcriptomic or proteomic analyses.

## Author contributions

Hernán López-Schier designed the study. Filipe Pinto-Teixeira, Mariana Muzzopappa, Alessandro Mineo, and Jim Swoger performed the experiments. James Sharpe and Jim Swoger designed the SPIM system. Hernán López-Schier, Filipe Pinto-Teixeira, Mariana Muzzopappa analyzed the data. Jim Swoger, James Sharpe, Hernán López-Schier, Filipe Pinto-Teixeira, Mariana Muzzopappa participated in designing and discussing the project. Hernán López-Schier wrote the paper with assistance from Filipe Pinto-Teixeira, Jim Swoger, and Mariana Muzzopappa.

### Conflict of interest statement

The authors declare that the research was conducted in the absence of any commercial or financial relationships that could be construed as a potential conflict of interest.

## Supplementary material

Supplementary Movie 1 **UHCPs division and planar cell inversion**. A 916' minute series of confocal images of a double-transgenic Tg[Cldnb:mGFP;SqET4] regenerating neuromast. Two identified, and labeled with a yellow and green dot, UHCP eventually divide into a pair of sister hair cells.

Supplementary Movie 2 **Three-dimensional imaging by SPIM**. A 2-channel 68 h maximum-value projection time-lapse recorded using SPIM, showing the deposition of 2 neuromasts and their subsequent development. Ath1 (showed in red) is expressed in hair-cells and can be detected in hair-cell progenitors a few hours before the neuromasts are deposited. atho1a:tdTomato is observed later in dividing, young and mature hair-cells. All the cell nuclei are stained in green (H2B:GFP RNA) and the lateral line system is highlighted in green with the Clndnb:mGFP reporter. The brightness of each image has been normalized independently, so that the tdTomato fluorescence at early time-points is visible.

## References

[B1] Behra M. Bradsher J. Sougrat R. Gallardo V. Allende M. L. Burgess S. M. (2009). Phoenix is required for mechanosensory hair cell regeneration in the zebrafish lateral line. PLoS Genet. 5:e1000455 10.1371/journal.pgen.1000455 19381250 PMC2662414

[B2] Bleckmann H. Zelick R. (2009). Lateral line system of fish. Integr. Zool. 4, 13–25 10.1111/j.1749-4877.2008.00131.x 21392273

[B3] Brigande J. V. Heller S. (2009). Quo vadis, hair cell regeneration? Nat. Neurosci. 12, 679–685 10.1038/nn.2311 19471265 PMC2875075

[B4] Brignull H. R. Raible D. W. Stone J. S. (2009). Feathers and fins: non-mammalian models for hair cell regeneration. Brain Res. 1277, 12–23 10.1016/j.brainres.2009.02.028 19245801 PMC2700174

[B5] Chen W. Jongkamonwiwat N. Abbas L. Eshtan S. J. Johnson S. L. Kuhn S. (2012). Restoration of auditory evoked responses by human ES-cell-derived otic progenitors. Nature 490, 278–282 10.1038/nature11415 22972191 PMC3480718

[B6] Chiu L. L. Cunningham L. L. Raible D. W. Rubel E. W. Ou H. C. (2008). Using the zebrafish lateral line to screen for ototoxicity. J. Assoc. Res. Otolaryngol. 9, 178–190 10.1007/s10162-008-0118-y 18408970 PMC2504598

[B7] Choo B. G. Kondrichin I. Parinov S. Emelyanov A. Go W. Toh W-C. (2006). Zebrafish transgenic enhancer TRAP line database (ZETRAP). BMC Dev. Biol. 6:5 10.1186/1471-213X-6-5 16478534 PMC1386650

[B8] Collado M. S. Burns J. C. Hu Z. Corwin J. T. (2008). Recent advances in hair cell regeneration research. Curr. Opin. Otolaryngol. Head Neck Surg. 16, 465–471 10.1097/MOO.0b013e32830f4ab5 18797290 PMC2692475

[B9] Corwin J. T. Oberholtzer J. C. (1997). Fish n' chicks: model recipes for hair-cell regeneration? Neuron 19, 951–954 10.1016/S0896-6273(00)80386-4 9390508

[B10] Dempsey W. P. Fraser S. E. Pantazis P. (2012). PhOTO zebrafish: a transgenic resource for *in vivo* lineage tracing during development and regeneration. PLoS ONE 7:e32888 10.1371/journal.pone.0032888 22431986 PMC3303793

[B11] Detrich H. W. 3rd. Westerfield M. Zon L. I. (2011). The zebrafish. Preface. Methods Cell Biol. 105, xxi–xxii 10.1016/B978-0-12-381320-6.00027-8 22043535

[B12] Dijkgraaf S. (1963). The functioning and significance of the lateral-line organs. Biol. Rev. Camb. Phil. Soc. 38, 51–105 10.1111/j.1469-185X.1963.tb00654.x 14027866

[B13] Edge A. S. Chen Z. Y. (2008). Hair cell regeneration. Curr. Opin. Neurobiol. 18, 377–382 10.1016/j.conb.2008.10.001 18929656 PMC5653255

[B14] Faucherre A. Baudoin J. P. Pujol-Martí J. López-Schier H. (2010). Multispectral four-dimensional imaging reveals that evoked activity modulates peripheral arborization and the selection of plane-polarized targets by sensory neurons. Development 137, 1635–1643 10.1242/dev.047316 20430744

[B15] Faucherre A. López-Schier H. (2011). Delaying Gal4-driven gene expression in the zebrafish with morpholinos and Gal80. PLoS ONE 6:e16587 10.1371/journal.pone.0016587 21298067 PMC3027692

[B16] Ghysen A. Dambly-Chaudiere C. (2007). The lateral line microcosmos. Genes Dev. 21, 2118–2130 10.1101/gad.1568407 17785522

[B17] Greger K. Swoger J. Stelzer E. H. (2007). Basic building units and properties of a fluorescence single plane illumination microscope. Rev. Sci. Instrum. 78, 023705 10.1063/1.2428277 17578115

[B18] Haas P. Gilmour D. (2006). Chemokine signaling mediates self-organizing tissue migration in the zebrafish lateral line. Dev. Cell 10, 673–680 10.1016/j.devcel.2006.02.019 16678780

[B19] Harris J. A. Cheng A. G. Cunningham L. L. MacDonald G. Raible D. W. Rubel E. W. (2003). Neomycin-induced hair cell death and rapid regeneration in the lateral line of zebrafish (*Danio rerio*). J. Assoc. Res. Otolaryngol. 4, 219–234 10.1007/s10162-002-3022-x 12943374 PMC3202713

[B20] Huisken J. Stainier D. Y. (2009). Selective plane illumination microscopy techniques in developmental biology. Development 136, 1963–1975 10.1242/dev.022426 19465594 PMC2685720

[B21] Huisken J. Swoger J. Del Bene F. Wittbrodt J. Stelzer E. H. (2004). Optical sectioning deep inside live embryos by selective plane illumination microscopy. Science 305, 1007–1009 10.1126/science.1100035 15310904

[B22] Kaufmann A. Mickoleit M. Weber M. Huisken J. (2012). Multilayer mounting enables long-term imaging of zebrafish development in a light sheet microscope. Development 139, 3242–3247 10.1242/dev.082586 22872089

[B23] Kelley M. W. (2003). Exposing the roots of hair cell regeneration in the ear. Nat. Med. 9, 1257–1259 10.1038/nm1003-1257 14520370

[B24] Kemp H. A. Carmany-Rampey A. Moens C. (2009). Generating chimeric zebrafish embryos by transplantation. J. Vis. Exp. pii:1394. 10.3791/1394 19617875 PMC2770904

[B25] Kettleborough R. N. Bruijn E. Eeden F. Cuppen E. Stemple D. L. (2011). High-throughput target-selected gene inactivation in zebrafish. Methods Cell Biol. 104, 121–127 10.1016/B978-0-12-374814-0.00006-9 21924159

[B26] Kros C. J. (2007). How to build an inner hair cell: challenges for regeneration. Hear. Res. 227, 3–10 10.1016/j.heares.2006.12.005 17258412

[B27] López-Schier H. (2004). Regeneration: did you hear the news? Curr. Biol. 14, R127–R128 10.1016/S0960-9822(04)00037-5 14986686

[B28] López-Schier H. (2010). Fly fishing for collective cell migration. Curr. Opin. Genet. Dev. 20, 428–432 10.1016/j.gde.2010.04.006 20452199

[B29] López-Schier H. Hudspeth A. J. (2006). A two-step mechanism underlies the planar polarization of regenerating sensory hair cells. Proc. Natl. Acad. Sci. U.S.A. 103, 18615–18620 10.1073/pnas.0608536103 17124170 PMC1656970

[B30] López-Schier H. St. Johnston D. (2001). Delta signaling from the germ line controls the proliferation and differentiation of the somatic follicle cells during Drosophila oogenesis. Genes Dev. 15, 1393–1405 10.1101/gad.200901 11390359 PMC312703

[B31] López-Schier H. Starr C. Kappler J. Kollmar R. Hudspeth A. J. (2004). Directional cell migration establishes the axes of planar polarity in the posterior lateral-line organ of the zebrafish. Dev. Cell 7, 401–412 10.1016/j.devcel.2004.07.018 15363414

[B32] Magilvy J. K. (1985). Quality of life of hearing-impaired older women. Nurs. Res. 34, 140–144 10.1097/00006199-198505000-00003 3846919

[B33] Megason S. G. Fraser S. E. (2007). Imaging in systems biology. Cell 130, 784–795 10.1016/j.cell.2007.08.031 17803903

[B34] Mizutari K. Fujioka M. Hosoya M. Bramhall N. Okano H. J. Okano H. (2013). Notch inhibition induces cochlear hair cell regeneration and recovery of hearing after acoustic trauma. Neuron 77, 58–69 10.1016/j.neuron.2012.10.032 23312516 PMC3573859

[B35] Moens C. B. Donn T. M. Wolf-Saxon E. R. Ma T. P. (2008). Reverse genetics in zebrafish by TILLING. Brief. Funct. Genomics Proteomics 7, 454–459 10.1093/bfgp/eln046 19028802 PMC2899843

[B36] Murphey R. D. Zon L. I. (2006). Small molecule screening in the zebrafish. Methods 39, 255–261 10.1016/j.ymeth.2005.09.019 16877005

[B37] Oshima K. Shin K. Diensthuber M. Peng A. W. Ricci A. J. Heller S. (2010). Mechanosensitive hair cell-like cells from embryonic and induced pluripotent stem cells. Cell 141, 704–716 10.1016/j.cell.2010.03.035 20478259 PMC2873974

[B38] Oshima K. Senn P. Heller S. (2009). Isolation of sphere-forming stem cells from the mouse inner ear. Methods Mol. Biol. 493, 141–162 10.1007/978-1-59745-523-7_9 18839346 PMC2861714

[B39] Parinov S. Kondrichin I. Korzh V. Emelyanov A. (2004). Tol2 transposon-mediated enhancer trap to identify developmentally regulated zebrafish genes *in vivo*. Dev. Dyn. 231, 449–459 10.1002/dvdy.20157 15366023

[B40] Pittet M. J. Weissleder R. (2011). Intravital imaging. Cell 147, 983–991 10.1016/j.cell.2011.11.004 22118457 PMC3824153

[B41] Raible D. W. Kruse G. J. (2000). Organization of the lateral line system in embryonic zebrafish. J. Comp. Neurol. 421, 189–198 10.1002/(SICI)1096-9861(20000529)421:2<189::AID-CNE5>3.3.CO;2-B 10813781

[B42] Rivolta M. N. (2013). New strategies for the restoration of hearing loss: challenges and opportunities. Br. Med. Bull. 105, 69–84 10.1093/bmb/lds035 23175701

[B43] Rivolta M. N. Li H. Heller S. (2006). Generation of inner ear cell types from embryonic stem cells. Methods Mol. Biol. 330, 71–92 10.1385/1-59745-036-7:71 16846017

[B44] Rompolas P. Deschene E. R. Zito G. Gonzalez D. G. Saotome I. Haberman A. M. (2012). Live imaging of stem cell and progeny behaviour in physiological hair-follicle regeneration. Nature 487, 496–499 10.1038/nature11218 22763436 PMC3772651

[B45] Rubel E. W. Furrer S. A. Stone J. S. (2013). A brief history of hair cell regeneration research and speculations on the future. Hear. Res. 297, 42–51 10.1016/j.heares.2012.12.014 23321648 PMC3657556

[B46] Santoriello C. Zon L. I. (2012). Hooked! Modeling human disease in zebrafish. J. Clin. Invest. 122, 2337–2343 10.1172/JCI60434 22751109 PMC3386812

[B47] Swoger J. Muzzopappa M. López-Schier H. Sharpe J. (2011). 4D retrospective lineage tracing using SPIM for zebrafish organogenesis studies. J. Biophotonics 4, 122–134 10.1002/jbio.201000087 20925108

[B48] Wibowo I. Pinto-Teixeira F. Satou C. Higashijima S. López-Schier H. (2011). Compartmentalized Notch signaling sustains epithelial mirror symmetry. Development 138, 1143–1152 10.1242/dev.060566 21343366

[B49] Williams J. A. Holder N. (2000). Cell turnover in neuromasts of zebrafish larvae. Hear. Res. 143, 171–181 10.1016/S0378-5955(00)00039-3 10771194

[B50] Xu Q. Stemple D. Joubin K. (2008). Microinjection and cell transplantation in zebrafish embryos. Methods Mol. Biol. 461, 513–520 10.1007/978-1-60327-483-8_35 19030820

